# Imaging and Gross Pathological Appearance of Changes in the Parasagittal Grooves of Thoroughbred Racehorses

**DOI:** 10.3390/ani11123366

**Published:** 2021-11-24

**Authors:** Georgina C. A. Johnston, Benjamin J. Ahern, Chiara Palmieri, Alex C. Young

**Affiliations:** School of Veterinary Science, The University of Queensland, Gatton 4343, Australia; gcajohnston@gmail.com (G.C.A.J.); b.ahern@uq.edu.au (B.J.A.); c.palmieri@uq.edu.au (C.P.)

**Keywords:** horse, diagnostic imaging, third metacarpal bone, parasagittal groove, fracture, subchondral bone, Thoroughbred, MRI, CT, pathology

## Abstract

**Simple Summary:**

Early detection of racehorses at risk of stress fracture is key to reducing the number of horses with catastrophic fractures while racing. Bone changes are often visible in the limbs of Thoroughbred racehorses in work, particularly in the fetlock region. However, it is currently unknown whether some of these changes indicate an impending fracture or are a healthy adaptation to high-speed exercise. This study looks at imaging and gross changes in a specific area (parasagittal grooves (PSGs) of the cannon bone) and the utility of X-ray, computed tomography (CT) and magnetic resonance imaging (MRI) to detect the changes. All fetlock joints were assessed from twenty horses that died during racing or training, including horses with and without fetlock fracture. Overall, X-ray was poor for detecting PSG changes. Some PSG changes on CT and MRI were common in Thoroughbred racehorses and possibly represent normal bone adaptation when seen in clinical cases. However, certain CT and MRI findings were more prevalent in horses with a fracture, possibly indicating microdamage accumulation and increased risk of fracture. Bilateral advanced imaging is recommended in clinical cases of suspected fetlock pathology.

**Abstract:**

(1) Background: Parasagittal groove (PSG) changes are often present on advanced imaging of racing Thoroughbred fetlocks and have been suggested to indicate increased fracture risk. Currently, there is limited evidence differentiating the imaging appearance of prodromal changes in horses at risk of fracture from horses with normal adaptive modelling in response to galloping. This study aims to investigate imaging and gross PSG findings in racing Thoroughbreds and the comparative utility of different imaging modalities to detect PSG changes. (2) Methods: Cadaver limbs were collected from twenty deceased racing/training Thoroughbreds. All fetlocks of each horse were examined with radiography, low-field magnetic resonance imaging (MRI), computed tomography (CT), contrast arthrography and gross pathology. (3) Results: Horses with fetlock fracture were more likely to have lateromedial PSG sclerosis asymmetry and/or lateral PSG lysis. PSG lysis was not readily detected using MRI. PSG subchondral bone defects were difficult to differentiate from cartilage defects on MRI and were not associated with fractures. The clinical relevance of PSG STIR hyperintensity remains unclear. Overall, radiography was poor for detecting PSG changes. (4) Conclusions: Some PSG changes in Thoroughbred racehorses are common; however, certain findings are more prevalent in horses with fractures, possibly indicating microdamage accumulation. Bilateral advanced imaging is recommended in racehorses with suspected fetlock pathology.

## 1. Introduction

The increasing use of advanced imaging modalities for the examination of the fetlock region in racing Thoroughbreds has enabled improved identification of pathologies in horses presenting for lameness localized to the fetlock region [1]. Interpretation of the imaging findings is complicated, however, by the fact that certain changes likely represent a normal adaptive response of bones and can be considered within normal limits for an athlete in work. Additionally, horses at risk of fetlock fracture may not display clinical signs such as lameness or fetlock joint effusion, further complicating the identification of these horses. There has been recent cadaveric research regarding the appearance of articular cartilage defects and associated subchondral bone changes on advanced imaging modalities [2,3,4]. However, at the time of writing, there is no solid evidence differentiating the imaging appearance of some pathologies from normal stress-related modeling in response to galloping. This makes it difficult to determine which of the subclinical abnormalities identified with advanced imaging truly represent a legitimate cause for concern.

Third metacarpal or metatarsal condylar fractures are a common type of fetlock fracture [5,6]. Current evidence suggests that condylar fractures are fatigue fractures resulting from microfracture coalescence at the distal third metacarpal articular surface, particularly at the parasagittal grooves, with subsequent crack propagation proximally [7,8,9]. Pre-existing subchondral bone and articular cartilage pathology are often present in this region in horses with condylar fractures and these changes have the potential to be detected with advanced imaging prior to catastrophic breakdown [10,11,12,13].

Bone and articular cartilage pathology, in the parasagittal grooves of the distal third metacarpal and metatarsal bones, is common in racing Thoroughbreds [14,15,16]. However, certain imaging findings in the parasagittal grooves, such as short tau inversion recovery (STIR) hyperintensity on MRI or subchondral bone lysis with surrounding sclerosis, may be indicative of an increased risk of impending condylar fracture [17,18,19]. A better understanding of the advanced imaging appearance of the parasagittal grooves of Thoroughbred racehorses in work is required in order to begin to determine the clinical significance of such findings and make evidence-based decisions on the prognosis and risk of fetlock fracture [20].

Magnetic Resonance Imaging (MRI) and Computed Tomography (CT) are both increasing in availability worldwide and have the ability to be performed standing. However, there are different diagnostic advantages to each. There have been a few studies to date that have compared CT and high-field MRI findings for the osseous structures of the equine fetlock [2,3,21], but there have been no comparable studies in Australian Thoroughbred racing populations or on the use of a low-field standing MRI with a focus on the parasagittal groove region.

This study aims to compare the ability of cadaver radiography, CT and low-field standing MRI to diagnose and characterize pathology in the parasagittal grooves of the distal third metacarpal/metatarsal bone. By comparing relevant pathological findings and their appearance/visibility with the various imaging modalities, we will gain further knowledge about the relative utility of each modality for the diagnosis of pathology in the parasagittal grooves. We hypothesize that (1) certain advanced imaging findings are more prevalent in horses with fetlock fracture, (2) radiography has poor lesion detection, and that CT and MRI are superior imaging modalities for parasagittal groove pathology depending on the different pathology present and (3) an oblique frontal condylar plane of cross-sectional image acquisition allows improved visualization and assessment of pathology in the parasagittal grooves when compared to frontal plane images.

## 2. Materials and Methods

Cadaver distal limbs were collected from racing Thoroughbreds in work that were euthanized or had died in south-east Queensland. Data was collected on the signalment of the horse, previous racing history, the type and location of fracture and racetrack location and surface conditions. The study received animal ethics approval from The University of Queensland Animal Ethics Committee (SVS/ANRFA/041/18) and consent was obtained for the use of each horse from the owner or trainer.

Limbs were removed from the horses at the proximal metacarpus/metatarsus and wrapped. All distal limbs were either refrigerated at 4 °C or frozen at −20 °C within a maximum of 12 h after death. All cadaver fetlocks underwent a complete radiographic, CT (plain CT and contrast arthrography), MRI and post-mortem examination. If refrigerated, limbs were examined within 24 h of refrigeration. If frozen, limbs were thawed at 20 °C for 12 h and imaged directly, post-thawing prior to a gross pathological examination.

A standard radiographic series of each fetlock region using digital radiography (DR) was acquired as per a typical radiographic examination performed by an equine racetrack veterinarian. Dorsopalmar/-plantar, lateromedial, dorsolateral-45°-palmaromedial/-plantaromedial oblique and dorsomedial-45°-palmaromedial/plantarolateral oblique projections of the limbs were obtained with the limbs held in a weight-bearing position. Limbs were held flexed for flexed lateromedial and flexed dorsopalmar/-plantar projections.

**Figure 1:**
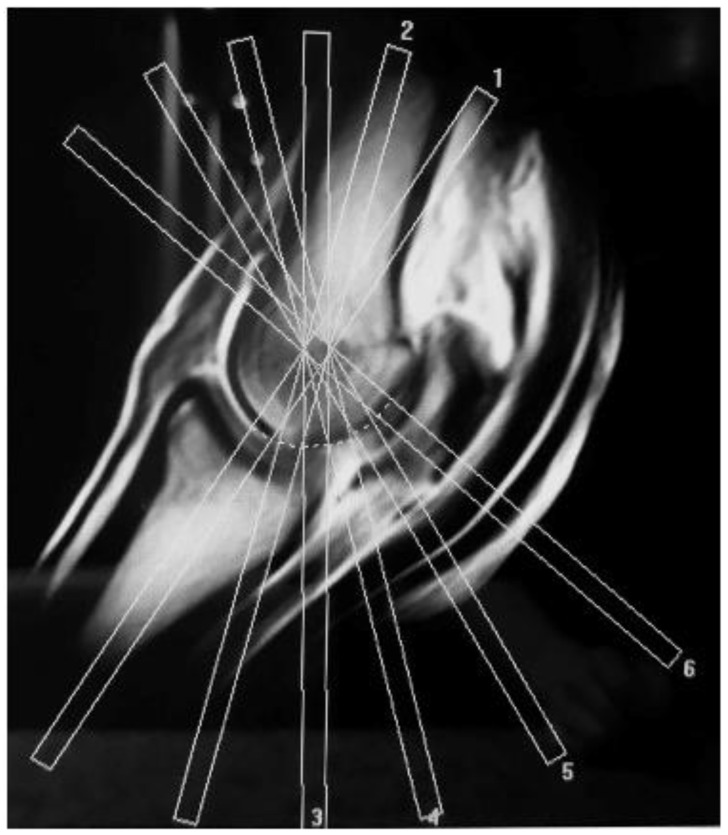
Sagittal MR image demonstrating the slice orientation for sequences in the condylar plane. The slices are placed in an oblique frontal orientation perpendicular to the palmar/plantar joint surface of the distal condyles.

MRI of the limbs was performed using a standardised protocol. MR images were acquired with a 0.27T standing MRI system (Hallmarq). A fetlock coil was used, and the leg was secured in a weight-bearing position within the magnet. T1-weighted (T1W) gradient recalled echo (GRE), T2-weighted (T2W) fast spin echo (FSE), T2*-weighted (T2*W) GRE and STIR FSE sequences were acquired in transverse, frontal and sagittal planes, aligned parallel and perpendicular to the long axis of the 3rd metacarpal/metatarsal bone. An additional T1W GRE oblique frontal (‘condylar’) sequence was acquired in sequential radiating slice planes perpendicular to the articular surface of the palmar/plantar 3rd metacarpal/metatarsal bone in order to optimally visualize palmar/plantar condylar lesions (Figure 1). For the STIR sequences, the inversion time (TI) was adjusted to optimize fat suppression for each leg.

CT examination of the limbs was performed using an Activion™ 16 Multislice system (Toshiba Medical Systems Corporation, Otawara, Japan) prior to and following the intra-articular injection of the fetlock joint with positive contrast media. Contiguous, transverse 0.5 mm collimated images of the fetlocks were acquired in soft tissue and bone algorithms from the level of the mid metacarpus/metatarsus to the mid proximal phalanx with the dorsal aspect of the limb orientated flat to the table. Using the palmaro-/plantarolateral intra-articular approach, 20 mL of diluted positive contrast media (Iohexol 300 mg/mL diluted in sterile saline 50:50) was injected into the fetlock joint following the initial image acquisition. The joint was flexed and extended 10 times to ensure complete contrast filling and the image acquisition was repeated. The purpose of the intra-articular contrast was to enable the visualization of the cartilage within the joint.

All images were simultaneously reviewed and graded by two of the investigators, a specialist in Veterinary Diagnostic Imaging (ACVR-EDI) (AY) and a resident in Equine Sports Medicine and Rehabilitation (GJ). Studies were viewed in “digital imaging and communications in medicine” (DICOM) format on an interactive DICOM viewer workstation (Osirix MD). CT images were viewed in MPR format in order to be able to orient slices as required to optimize the visualization of pathology. A modified grading system based on previous relevant publications was used to characterize the presence of changes in the parasagittal grooves of the distal third metacarpal/metatarsal bone and surrounding subchondral bone [2,4].

On DR, subchondral bone lucency in the medial and lateral parasagittal grooves was defined as decreased bone mineral opacity and graded as absent (0) or present (1).

**Figure 2:**
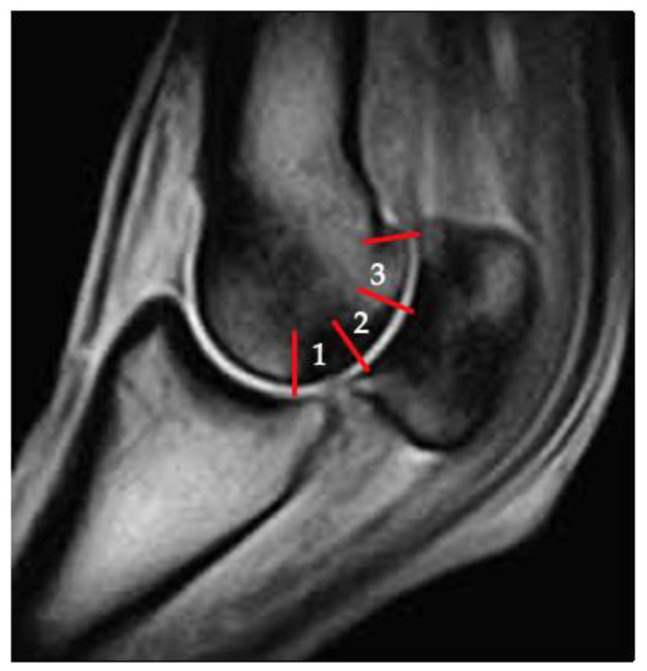
Sagittal MR image demonstrating grading zones 1, 2 and 3 of the palmar and plantar condyles.

Sclerosis of the subchondral and adjacent medullary bone of the parasagittal grooves was graded independently on all imaging modalities. Sclerosis was defined on DR as increased mineral opacity, on CT as increased medullary bone attenuation and on T1W, T2W and T2*W MRI sequences as reduced medullary bone signal intensity. For DR, sclerosis was graded as absent (0) or present (1) in the medial and lateral parasagittal grooves. For CT and MRI, the severity of sclerosis was graded according to the estimated volume extent of changes. Grade 0 is none, Grade 1 is mild (<1/3^rd^ of the subregion), Grade 2 is moderate (1/3^rd^ to 2/3^rds^ of the subregion) and Grade 3 is severe (>2/3^rds^ of the subregion). The zones (1, 2 or 3) of the palmar/plantar distal metacarpus/metatarsus, in which the sclerosis was present, were documented (Figure 2), medially and laterally, in both frontal and condylar planes. Sclerosis was recorded as being centered on the parasagittal groove or centering on the condyle. On MRI, the sequences in which the sclerosis was visible were recorded (T1W frontal, T2W frontal or T1W condylar).

For MR images, the lateral and medial parasagittal grooves were graded for the presence of an abnormality or defect (0 = absent, 1 = present). This was further defined according to the presence of subchondral bone defects or fissures, subchondral bone lucency or suspected cartilage loss. The sequences in which the abnormality was visible were recorded. STIR hyperintensity of the medial and lateral parasagittal grooves was graded as absent (0) or present (1) and as centered on the parasagittal groove or centering on the condyle.

For CT images, the lateral and medial parasagittal grooves were graded for the presence of an abnormality or defect (0 = absent, 1 = present). This was further defined with respect to the presence of cartilage thickening, irregularity and hypoattenuation, cartilage loss, subchondral bone defect or fissure, subchondral bone irregularity and subchondral bone lucency. Subchondral bone lucency was defined as decreased subchondral/trabecular bone attenuation. The orientation (frontal or oblique frontal/‘condylar’) in which the abnormality was visible was recorded. Images both with and without contrast arthrography were assessed in order to grade each parameter.

A post-mortem examination was performed by one investigator (GJ) directly following the completion of the imaging. All fetlock joints were dissected using a standardised method of disarticulation. Joint surfaces were macroscopically examined and digitally photographed for records and grading. All the digital photographs were graded by one investigator (GJ) within a one-day period to improve consistency between the limbs. Gross pathology grading was performed while blinded to the identity of the horse and the imaging findings. The lateral and medial parasagittal grooves were each graded for cartilage fissures, not present (grade 0) or present (grade 1). If a fissure was present, further grading was performed: 1 = mild (faint groove with intact cartilage visible along the length), 2 = moderate (well-defined groove with partial thickness split in cartilage) and 3 = severe (well-defined groove with full-thickness cartilage split). The previously described zones 1, 2 and 3 (Figure 2), in which the fissures were present, was also documented. This grading system was used due to the high repeatability shown in previous studies using similar grading systems [22]. 

Targeted histopathology was performed on parasagittal grooves that had subchondral bone fissures detected on CT in an attempt to differentiate true bone fissures from vasculature in this region. Bone specimens (1 × 2.5 cm) were collected from the affected areas. Tissue samples were submitted in 10% neutral buffered formalin and demineralized in 15% formic acid, prior to trimming. Tissues were embedded in paraffin, sectioned at 5 μm and stained with hematoxylin and eosin. Microscopic examination was performed by a specialist pathologist (CP).

### Data Analysis

An investigation of imaging changes specifically located in the parasagittal groove or with intraosseous fetlock STIR hyperintensity has not previously been performed in the cadaver limbs of racing Thoroughbreds. Therefore, sample size estimates were based on results comparing different fetlock pathologies across radiography, CT and MRI, and the average sensitivity and specificity estimates for each imaging modality in previously published papers [3,21].

Sample size estimates were conducted using a custom commercial software package designed for power analysis and sample size (PASS, www.ncss.com, accessed on 20 October 2019). Input assumptions included alpha (0.05) and beta (0.1). A sample size of 72 fetlock joints achieved 90% power to detect a difference of −3.1 between the actual mean of 5.0 and the null hypothesized mean of 8.1 with a known standard deviation of 8.1 and with a significance level (alpha) of 0.050 using a two-sided one-sample z-test.

These findings suggest that our sample size of 80 fetlock joints was likely to produce results that were statistically meaningful. Each PSG change was compared across all imaging modalities and, if applicable, with gross pathology. Parasagittal groove findings and their appearance/visibility with the different imaging modalities were compared to assess the relative diagnostic use of each modality.

Limbs were divided into groups for analysis by (1) reason for death: fracture in the fetlock region (FF), fracture distant to the fetlock region (OF) and non-fracture control (NF); (2) age: adult (older than three years old) and juvenile (two- and three-year-olds); (3) contralateral limb to fracture (fetlock and other) group with NF group as control. 

Data was further divided on a limb level into medial and lateral parasagittal grooves. Imaging and gross appearance were correlated with horse data to look for trends or prodromal factors related to fetlock fracture. All analyses performed at the horse level were performed using Generalized Mixed Model analysis (GLMM) using binomial distribution. The horse ID was considered as a random effect to account for the clustering effect of legs belonging to the same horse. The coefficient estimates, 95% Confidence Intervals (CI) and *p*-values are presented.

Descriptive statistics for continuous variables were reported using means and standard deviations or median and inter-quartile ranges when distribution was skewed. Frequencies and percentages were reported for categorical variables. A Fisher’s exact test was performed to test for associations between two categorical variables. When the relationship was significant and one of the variables had more than two categories, post-hoc pairwise Fisher’s exact tests were performed, and all *p*-values were adjusted for multiple testing using the FDR correction. All analyses were performed using the R statistical software. *p*-values < 0.05 were considered significant and *p*-values from 0.05 to 0.08 were used to signify a trend.

## 3. Results

### 3.1. Horse Data

Eighty limbs from 20 Thoroughbred horses in race work in south-east Queensland were collected from February 2018 to June 2020. Horses were collected from Eagle Farm, Gold Coast, Doomben, Toowoomba and Deagon racetracks with no racetrack overrepresented. The horses were an average age of 3.5 years old (median 3, range 2–6). Seven of the horses were females, 10 were geldings and three were entire males. Eight died while racing, nine while training and three under other circumstances (two in racing stables and one in recovery from general anesthesia).

When divided by reason for death, the FF group included five horses (four biaxial sesamoid bone fractures and one transverse distal third metacarpal bone fracture), the OF group included six horses (three tibial fractures, two humeral fractures and one proximal third metatarsal fracture) and the NF group included nine horses (seven had sudden death while racing or training and two were euthanized for colic). When divided by age, the adult group had seven horses and the juvenile group had 13 horses. There were 11 limbs in the contralateral limb to fracture group.

Four limbs with catastrophic fetlock fracture had extensive articular and subchondral bone trauma to the fetlock post-fracture and were therefore excluded from the analysis. Four additional limbs (from one horse in the NF group) were excluded from the STIR hyperintensity data due to poor fat suppression on MRI.

### 3.2. Sclerosis of the Subchondral and Adjacent Medullary Bone of the PSGs

Overall, subchondral/medullary bone sclerosis was present on CT in 89% of PSGs (135/152), on MRI in 70% (106/152) and on radiography in 11% (16/152). Overall, the mean grade of severity was 1.17 (range 0–3) in the medial PSGs and 0.89 (range 0–3) in the lateral PSGs. The prevalence of lateral and medial PSG sclerosis on radiography, CT and MRI for the FF, OF and NF groups, for the juvenile and adult groups and for the contralateral limb group, is shown in Table 1.

The difference in sclerosis grades between lateral and medial PSGs on CT was calculated. In the FF group, 12% of limbs (2/16) had symmetry between lateral and medial condylar sclerosis grade compared to 71% (17/24) in the OF group and 83% (30/36) in the NF group. The proportion of limbs with sclerosis asymmetry was significantly higher in the FF group compared to the NF group (Fisher’s exact test, *p* = 0.001). However, in the GLMM, no significant increased likelihood of experiencing fetlock fracture was identified in the horses with sclerosis asymmetry.

When considering Grade 0/1 and Grade 2/3 as pooled, the FF group had symmetry between lateral and medial condylar sclerosis grades in 44% of limbs (7/16) compared to 79% (19/24) in the OF group and 92% (33/36) in the NF group. Of the FF group with sclerosis asymmetry, 33% (3/9) had lateral grade two or three and medial grade zero or one and 67% (6/9) had medial grade two or three and lateral grade zero or one.

**Figure 3:**
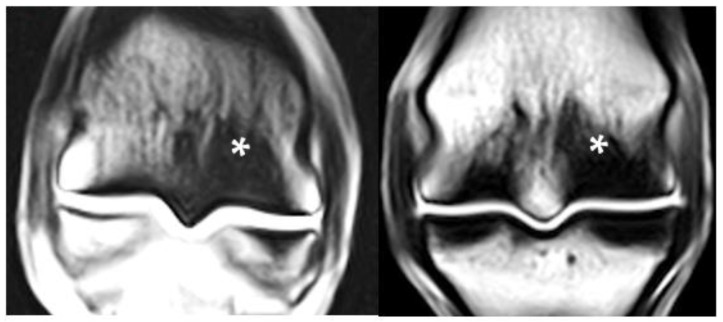
Examples of mediolateral asymmetry in condylar and parasagittal groove sclerosis on T1W frontal MR images in the contralateral limbs of a 2-year-old Thoroughbred filly (**left**) with right fore biaxial sesamoid fracture and a 3-year-old Thoroughbred gelding (**right**) with left fore biaxial sesamoid fracture during racing. Note the greater severity and triangular shape of the sclerosis medially (*). Lateral is left in the images.

The proportion of asymmetry between lateral and medial condylar sclerosis was significantly higher in the contralateral limb to fracture compared to the control group (Fisher’s exact test, *p* = 0.011) with 45% (5/11) of limbs with asymmetry in the contralateral limb to fracture compared to 8% (3/36) in the control group. Limbs contralateral to fracture had a trend towards increased likelihood of asymmetry between lateral and medial condylar sclerosis compared to the control group (Figure 3; GLMM coefficient estimate 1.30, 95% CI [0.07, 2.66], *p* = 0.06).

The grade of POD was significantly associated with the grade of PSG sclerosis. In the lateral condyle in the frontal plane (*p* = 0.002), the condylar plane (*p* = 0.005) and in the medial condyle in the frontal plane (*p* = 0.023) POD grade two or three was significantly associated with grade two or three PSG sclerosis in zones one + two or zone one only.

The prevalence of sclerosis on CT and MRI in the condylar and frontal planes is shown in Table 1. Changing from frontal plane to condylar plane for the detection of sclerosis in zone one increased the agreeance between MRI and CT from 57% to 64% for the lateral PSG and from 72% to 83% for the medial PSG.

### 3.3. PSG Subchondral Bone Lysis

**Figure 4:**
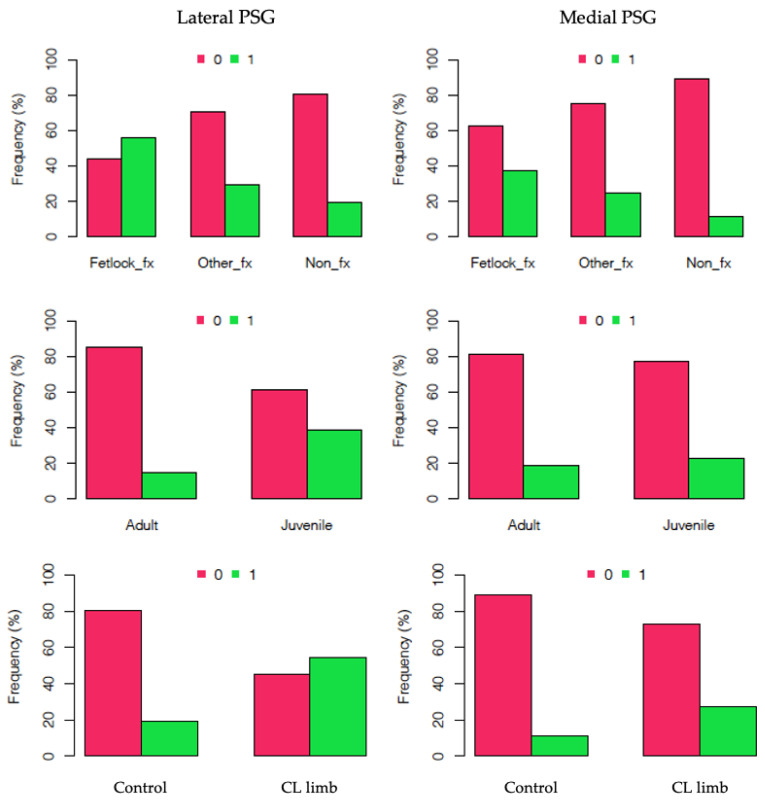
Bar graphs showing the frequency of subchondral bone lysis on CT in the lateral (**left**) and medial (**right**) parasagittal grooves (PSGs) divided by fetlock fracture (fetlock_fx), other fracture (other_fx) and non-fracture (non_fx) groups (**top**); adult and juvenile groups (**middle**) and contralateral (CL) limb to fracture and control groups (**bottom**). 0 = no lysis and 1 = lysis present.

Overall, SCB lysis was present in 29% (23/76) of lateral PSGs and 20% (16/76) of medial PSGs. In the FF group, 47% (15/32) of PSGs had SCB lysis, compared to 27% (13/48) in the OF group and 15% (11/72) in the NF group (Figure 4). Horses with lateral PSG lysis had a trend towards increased likelihood of fracture (fetlock and elsewhere) compared to those without lateral PSG lysis (coefficient estimate 1.22, 95% CI [−0.109, 2.57], *p* = 0.07). Split by age, the juvenile group had SCB lysis in 31% (30/98) of PSGs compared to 17% (9/54) in the adult group (Figure 4). Juvenile horses had a trend towards increased likelihood of experiencing PSG lateral lysis in at least one of their legs compared to adult horses (coefficient estimate 1.52, 95% CI [−0.13, 3.65], *p* = 0.08).

**Figure 5:**
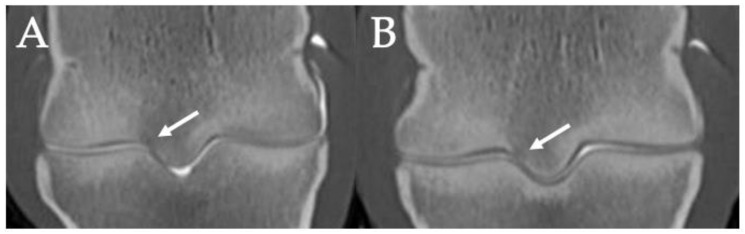
Frontal CT images of focal subchondral bone lysis (white arrows) in the lateral parasagittal grooves of the left (**A**) and right (**B**) hindlimbs of a 3-year-old Thoroughbred colt with left tibial fracture while racing. Lateral is left in the images.

SCB lysis was present in 41% (9/22) of PSGs in the contralateral limbs to fracture, of which two thirds (6/9) were in the lateral PSG (Figure 5), and in 15% (11/72) of PSGs in the control group (Figure 4).

Using CT as the gold standard, MRI detected 0 (0%) of 23 lateral PSGs and 16 medial PSGs with SCB lysis (true positives). Of 53 lateral PSGs and 60 medial PSGs with no SCB lysis on CT, 111 (98%) were also recorded as no lysis on MRI (true negatives) and 2 (2%) were recorded as having a lysis on MRI (false positives). The false positives both had grade two or three cartilage lesions on gross pathology.

### 3.4. PSG Subchondral Bone Defects

Overall, SCB defects were detected on CT in 57% (86/152) of PSGs, on MRI in 47% (72/152) and on radiography in 25% (38/152). Prevalence of lateral and medial PSG SCB defects on radiography, CT and MRI for the FF, OF and NF groups, for the juvenile and adult groups and for the contralateral limb group, is shown in Table 2. Medial and lateral distribution of PSG SCB defects was not statistically significantly different for reason for death (*p* = 0.707), or contralateral limb group compared to controls (*p* = 1.000).

Using CT as the gold standard, false positives and false negatives on MRI were high (see Tables S1 and S2, Supplementary Materials). Most of the false positives for lateral (4/5, 80%) and medial (8/11, 73%) PSG SCB defects on MRI had a cartilage defect grade of two or three. There was a significant positive association between the presence of a subchondral bone defect and a gross pathology grade two or three cartilage lesion in the parasagittal grooves (*p* < 0.001).

Most false positives were detected on T1W frontal (81%), followed by T2W frontal (31%), T1W condylar (25%), T1W transverse (25%) and T2*W frontal (19%). Most true positives were detected on T1W frontal (96%), T1W condylar (59%) and T1W transverse (57%), followed by T2W frontal (30%) and T2*W frontal (30%). Subchondral bone defects on CT were best visualized on the parasagittal plane with contrast arthrography.

### 3.5. PSG Cartilage Defects

Overall, cartilage lesions in the parasagittal grooves were detected on gross pathology in 94% (143/152) of PSGs, on CT in 90% (137/152) and on MRI in 75% (118/152). Cartilage lesions on gross pathology were present in 89% (71/76) of medial and 90% (72/76) of lateral PSGs. The prevalence and grade distribution of medial and lateral PSG cartilage lesions on gross pathology, CT and MRI for the FF, OF and NF groups, for the juvenile and adult groups and for the contralateral limb group, is shown in Table S3, Supplementary Materials.

The difference in grade of severity of PSG cartilage lesions on gross pathology was calculated between medial and lateral PSGs. In the FF group, 63% (10/16) of medial PSG cartilage defects had a higher grade of severity than lateral PSG cartilage defects, compared to 20% (5/24) in the OF group and 6% (2/36) in the NF group.

Using gross pathology as the gold standard, contrast CT had a sensitivity of 0.929 and a specificity of 0.325, and MRI had a sensitivity of 0.812 and a specificity of 0.775 for detecting cartilage defects in the parasagittal grooves.

**Figure 6:**
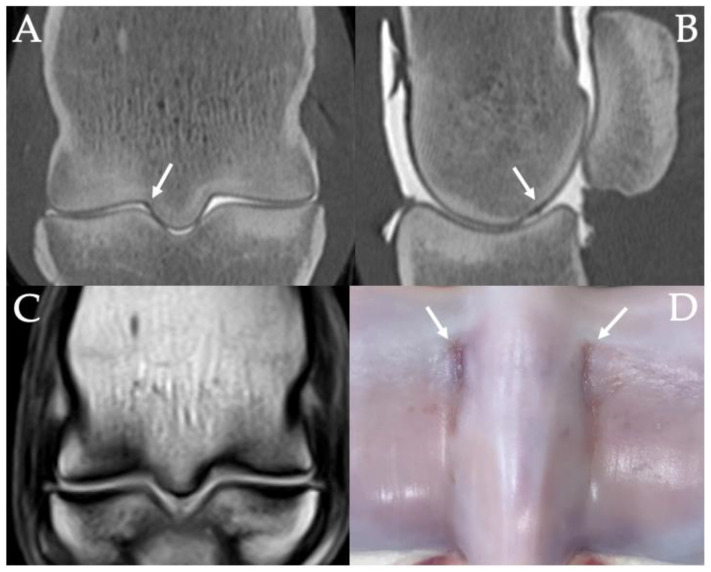
Contrast arthrography CT images of the distal third metacarpal bone in frontal (**A**) and sagittal (**B**) planes of the right hind fetlock of a 4-year-old Thoroughbred gelding with left tibial fracture during race training. Cartilage surface irregularity, thickening and hypoattenuation are indicated with white arrows. A frontal plane T1W image of the same limb (**C**) shows how the presence of cartilage defects can complicate the interpretation of the subchondral bone on MRI, potentially leading to a false-positive diagnosis of subchondral bone defects. Image (**D**) shows the articular cartilage defects in the parasagittal grooves of the distal third metacarpal bone on post-mortem. Lateral or dorsal are to the left in all images.

The majority of the PSG cartilage abnormalities detected on CT had focal surface irregularities, thickening and/or hypoattenuation of the cartilage (124/137, 91%) (Figure 6). Partial or complete cartilage loss was detected on CT in 9% (13/152) of parasagittal grooves, of which 92% (12/13) were grade two or three on gross pathology. Cartilage loss was found in 11% (8/76) of medial PSGs and 7% (5/76) of lateral PSGs.

### 3.6. PSG STIR Hyperintensity

**Figure 7:**
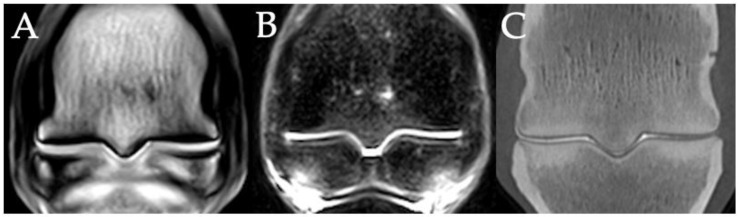
MRI image of STIR hyperintensity in the parasagittal grooves (**B**) of the right fore fetlock of a 4-year-old Thoroughbred gelding with a left tibial fracture during race training. The only other parasagittal groove abnormality detected on MRI (**A**) or CT (**C**) was mild cartilage fibrillation. Lateral is left in the images.

Overall, STIR hyperintensity was present in 44% of lateral PSGs (30/68) and in 29% of medial PSGs (20/68). The prevalence of STIR hyperintensity in the lateral and medial PSGs for the FF, OF and NF groups, for the juvenile and adult groups and for the contralateral limb group, is shown in Table 3. In three limbs, PSG STIR hyperintensity was detected on MRI with no associated findings detected in the parasagittal grooves on CT (Figure 7).

The presence of SCB lysis on CT was found to be significantly associated with STIR hyperintensity in the lateral (*p* = 0.04) and medial (*p* = 0.02) PSGs. No significant association was found between STIR hyperintensity and the presence of an SCB defect on CT in the lateral *(p =* 0.09) or medial (*p* = 0.11) PSGs.

All PSGs with grade three sclerosis on CT also had STIR hyperintensity on MRI, regardless of zone or imaging plane (17/17, 100%). In lateral zone one, there was a significantly higher prevalence of STIR hyperintensity on MRI in PSGs with grade three sclerosis (6/6, 100%) compared to PSGs with no sclerosis (3/16, 19%) (*p* = 0.007). There was a trend towards a similar difference between PSGs with sclerosis grade one and three (*p* = 0.065) with only 42% (17/40) of grade one having STIR hyperintensity compared to 100% in grade three. In medial zone two, there was a significantly higher prevalence of STIR hyperintensity in PSGs with grade three sclerosis (4/4, 100%) than PSGs with grade one sclerosis (10/48, 21%) (*p* = 0.022). Similar differences in presence of STIR signal between sclerosis grades were apparent for medial zone one and medial and lateral zone two; however, after adjustment for multiple testing, none of the comparisons was significant.

### 3.7. Combinations of PSG Pathology

Combinations of PSG pathologies were investigated in relation to the reason for death. For combinations of lysis and sclerosis, grade two or three sclerosis was found in conjunction with SCB lysis in 44% (7/16) of PSGs in the FF group and 17% (10/60) of PSGs in the combined OF + NF group. In contrast to this, grade two or three sclerosis without lysis was found in 19% (3/16) of PSGs in the FF group and 58% (35/60) of PSGs in the combined OF + NF group. However, with the GLMM, the presence of combined SCB sclerosis and SCB lysis on CT examination was not found to be significantly associated with a fracture.

The combination of a SCB defect and lysis on CT was found in 44% (7/16) of lateral PSGs in the FF group, in 29% (7/24) of the OF group and in 14% (5/36) of the NF group. The combination of grade two or three subchondral bone sclerosis, lysis and defect on CT with STIR hyperintensity on MRI was found in 29% of the lateral and 21% of the medial PSGs of the FF group, in 18% and 9% of the OF group and 3% and 0% of the NF group. However, the numbers were too low to detect statistical significance.

### 3.8. Fetlocks with Possible Fracture Pathology

**Figure 8:**
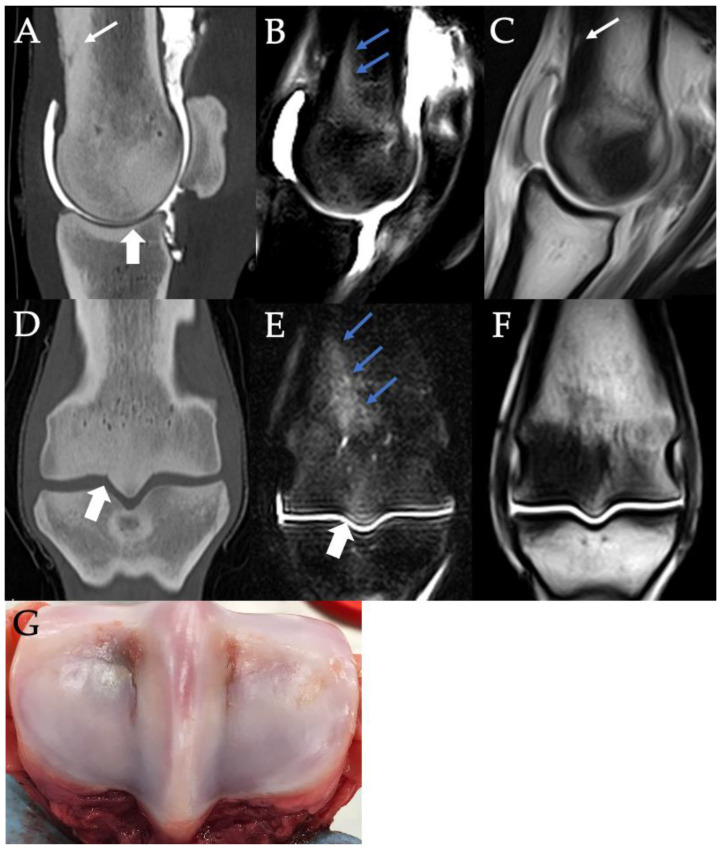
Contrast arthrography CT (**A**,**D**), STIR (**B**,**E**) and T1W (**C**,**F**) images in the sagittal (**A**–**C**) and frontal (**D**–**F**) planes of the right fore fetlock from a 3-year-old Thoroughbred colt in race training, euthanized for a left fore transverse distal third metacarpal bone fracture. There is a saucer-shaped, incomplete fracture line within the thickened dorsal cortex of the third metacarpus (small white arrows) with associated irregular new bone/callus at the dorsal to dorsolateral distal diaphysis (**A**). There is severe STIR hyperintensity associated with the dorsal cortical fracture as well as coursing obliquely in a proximolateral to distomedial orientation (small blue arrows). Markedly asymmetrical sclerosis is present between the lateral and medial condyles (**D**,**F**) with a possible lateral PSG fissure (white arrowheads) and lateral PSG STIR hyperintensity. Image (**G**) shows the articular surface and lateromedial asymmetry in pathology of the distal third metacarpal bone on post-mortem. Lateral or dorsal are left in all images.

**Figure 9:**
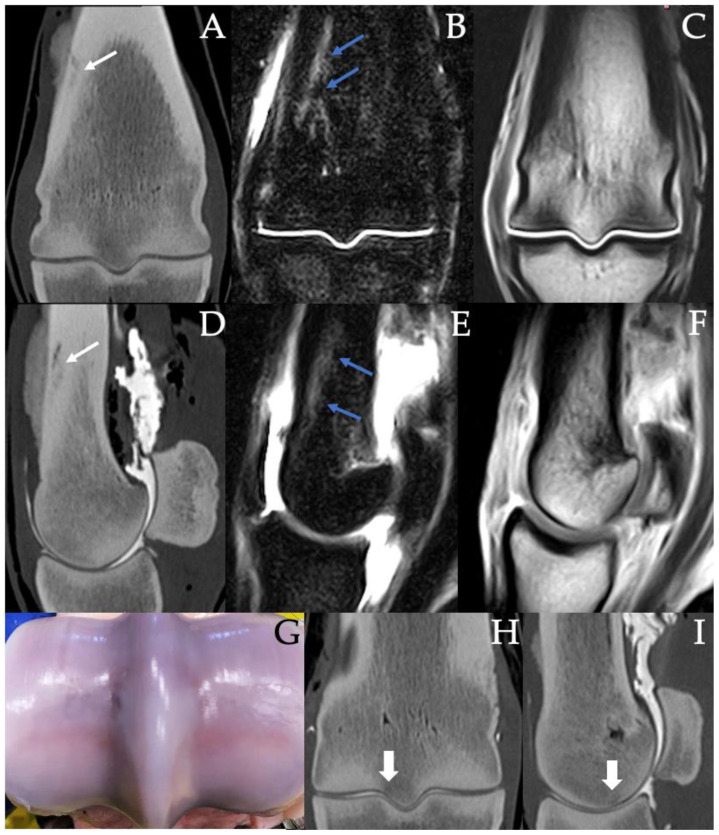
Contrast arthrography CT (**A**,**D**,**H**,**I**), STIR (**B**,**E**) and T1W (**C**,**F**) images in the frontal (**A**–**C**) and sagittal (**D**–**F**) planes of the right fore fetlock of a 3-year-old Thoroughbred gelding, euthanized for left fore biaxial sesamoid fracture while racing. Note the incomplete fracture line (small white arrows) and irregular periosteal new bone/callus at the dorsolateral distal diaphyseal cortex of the third metacarpus. Severe STIR hyperintensity was present in the medullary bone of the distal dorsolateral diaphysis (small blue arrows) with irregular to linear regions of STIR hyperintensity coursing obliquely between it and the axial distal physeal scar. Corresponding linear T1W hypointensities were visibly coursing obliquely through this region on the frontal T1W sequence (**C**). No STIR hyperintensity or fracture line was visible distal to the distal physeal scar; however, there was increased visibility of the physeal vasculature and focal osteolysis associated with the lateral PSG (white arrowheads). Image (**G**) shows the articular surface of the distal third metacarpal bone on post-mortem. Note the focal lateral PSG cartilage lesion. Lateral or dorsal are left in the images.

**Figure 10:**
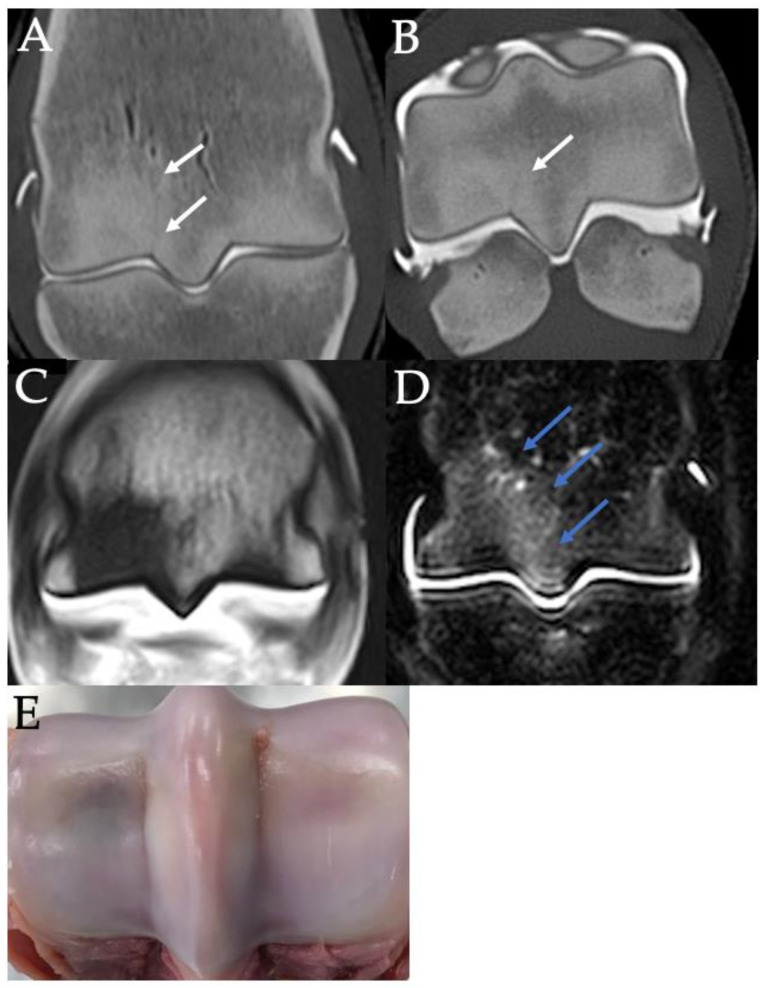
Contrast arthrography CT images in the frontal (**A**) and sagittal (**B**) planes and T1W frontal (**C**) and STIR frontal (**D**) images of the left hind fetlock from a 3-year-old Thoroughbred colt in race training, euthanized for a transverse distal third metacarpal bone fracture of the left forelimb. Note the marked asymmetry in the plantar condylar sclerosis with associated STIR hyperintensity (small blue arrows). This was suspected to be, in part, attributable to the presence of a severe POD lesion laterally. A lucent line is visible in the lateral PSG on CT possibly indicating an incomplete lateral condylar fracture/fissure or enlarged blood vessel (small white arrows). Image (**E**) shows the articular surface and asymmetrical pathology of the distal third metacarpal bone on post-mortem, with greater severity of parasagittal groove cartilage lesion medially and greater severity of POD laterally. Lateral is to the left in all images.

**Figure 11:**
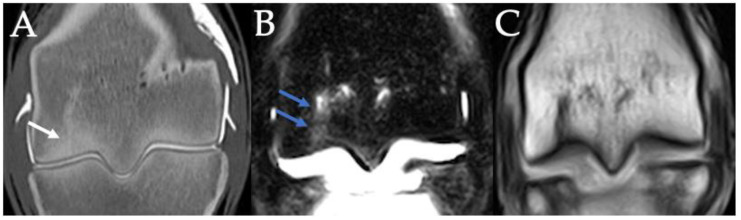
Contrast arthrography CT (**A**), STIR (**B**) and T1W (**C**) images in the frontal plane of the right hindlimb fetlock of a 3-year-old Thoroughbred colt with left tibial fracture while racing. Note the linear lucency extending between the subchondral bone and physeal scar (small white arrow) with associated marked, linear STIR hyperintensity (small blue arrows). There was concurrently increased visualization of the vasculature at the distal physeal scar. An incomplete fracture/fissure or enlarged vascular channel was considered possible in this case.

Eight horses (11 limbs) had pathology suggestive of incomplete fracture of the third metacarpal or metatarsal bone (Figure 8, Figure 9, Figure 10 and Figure 11), with some horses having possible fracture in more than one limb. In seven cases (7/11, 64%), these limbs were contralateral to a fractured limb (fetlock or elsewhere) (Figure 8 and Figure 9). Six limbs had evidence of regional periosteal new bone/callus formation.

Six of the limbs were considered to have possible, early non-displaced, incomplete fracture/fissure of the distal third metacarpal/metatarsal bone due to the presence of a narrow, linear lucency at the level of either a PSG or a POD lesion on CT examination. Four of these cases had associated STIR hyperintensity at the location of the suspected fracture (Figure 10 and Figure 11). These cases included horses from the FF, OF and NF groups. Although the presence or absence of regional STIR hyperintensity and/or possible callus formation was considered when assessing linear lucencies in these fetlocks, it was not possible to differentiate incomplete fracture/fissure lines from enlarged vascular channels with confidence in some of these cases.

### 3.9. Histopathology

Two bone sections from parasagittal grooves with suspected SCB fissures were processed for histological examination. Despite the use of transverse and longitudinal slices through the area of interest, the histopathological examination was unsuccessful in identifying subchondral bone fissures or blood vessels in the parasagittal grooves. Hence, the two could not be definitively distinguished in this study.

## 4. Discussion

Pathology in the parasagittal grooves of the third metacarpal/metatarsal bone is mostly thought of in the context of condylar fracture, and certain changes in the parasagittal grooves are thought to represent prodromal indicators of fracture in this location [10,16,17,23,24]. This study investigates the imaging appearance of changes in the parasagittal grooves of fetlocks in a specific Thoroughbred racehorse population using radiography, contrast CT arthrography and low-field MRI. The findings were investigated on a horse, limb and PSG level, both as singular observations and in specific combinations, with the goal of identifying any changes that may have a positive association with fetlock fracture.

None of the catastrophic fetlock fractures in this study resulted from condylar fracture. This study and other research performed in south-east Queensland at a similar time show that biaxial proximal sesamoid bone fractures are a more common cause of catastrophic fetlock fracture in this population of Thoroughbreds in work, with condylar fractures proving less common [25]. Despite this, all the fetlocks in this study had CT and/or low-field MRI evidence of parasagittal groove cartilage or bone changes, ranging in severity from mild sclerosis to subchondral bone defects, lysis and STIR hyperintensity. This suggests that some of these findings, though likely indicative of parasagittal groove pathology, may not represent prodromal change specific for condylar fracture. It is possible that certain parasagittal groove findings may represent prodromal changes indicative of skeletal overload and/or increased overall fracture risk, either in other regions of the fetlock or elsewhere in the appendicular skeleton.

### 4.1. Relevance to Fracture Risk

Certain advanced imaging findings in this study were found more commonly in the parasagittal grooves of horses with fracture (FF and OF groups), and if seen may indicate the presence of focal remodeling and sites of microdamage accumulation.

In this study, horses with fetlock fracture were more likely to have lateromedial PSG sclerosis asymmetry compared to horses with death from non-musculoskeletal reasons; however, statistical significance for this was not reached in the mixed model analysis. Asymmetrical sclerosis was commonly more severe medially and often triangular in shape, with the greatest region extending proximally to the axial distal physeal scar (Figure 3).

Lateromedial asymmetry in pathology has previously been described in association with biaxial proximal sesamoid bone fracture, with focal lesions on the medial proximal sesamoid bone indicating an increased risk of fracture in this location [26,27]. It is possible that a higher grade of sclerosis in the medial PSGs could be linked to the etiology of proximal sesamoid bone fractures, which are thought to initiate medially [26,28], but this requires further research. Additionally, horses at risk of lateral condylar fracture have been shown to have increased PSG pathology in the lateral PSG compared to the medial PSG [24,29]. A study looking at force distribution in limbs with and without lateral condylar fracture found that stresses were found to concentrate at the transverse ridge in horses without condylar fracture, while in limbs with condylar fracture and in their contralateral limbs, the highest stress was found in the lateral parasagittal groove [30]. In another study, STIR hyperintensity and T1W and T2*W hypointensity in a triangular shape at the lateral palmar condyle was associated with a lateral condylar fracture [31]. In the current study, the presence of asymmetrical sclerosis suggests an overall increase in fracture risk but not necessarily at the parasagittal groove.

Abnormal or altered distribution of joint contact forces may influence asymmetry of PSG and condylar sclerosis in the fetlock. However, research into what is normal and abnormal in the distribution of forces within the fetlock joint in Thoroughbred racehorses is still speculative and remains to be fully determined in vivo [32]. Horses may experience greater medial or lateral fetlock loading due to inherent factors such as limb conformation or shape and size of the distal metacarpus [33,34] or may have changed or abnormal loading due to osteoarthritis [35], other fetlock pathology, or musculoskeletal pain elsewhere.

From our study population and findings, it is hypothesized that the asymmetry in sclerosis between PSGs and condyles could indicate abnormal or changed patterns of force distribution through the entire fetlock joint, not just at a condylar level. Excessive loading or changes in load direction at injury predilection sites, such as the proximal sesamoid bones, could lead to focal microdamage accumulation and increased risk of subsequent fracture.

Four cases of incomplete condylar fractures occurring through POD lesions were suspected within the OF and NF groups in this study population. The suspected fracture lines were identified on CT as short, incomplete linear lucencies at the palmar/plantar articular surface of high-grade POD lesions centered in the condyle, not in the parasagittal groove. Previous research suggests that a substantial number of lateral condylar fractures originate in the mid palmar/plantar condyle, abaxial to the parasagittal groove; however, these studies do not document concurrent POD lesions in the affected condyles [29,36]. There is unpublished research from the University of Pennsylvania suggesting this appearance is consistent with an incomplete condylar fracture configuration detectable on standing CT [37]; however, we hypothesize that these linear lucencies could also represent enlarged vascular channels associated with POD pathology.

The contralateral limbs to fracture (fetlock or elsewhere) commonly had changes suggestive of focal remodeling or microdamage accumulation that were more severe than the other limbs in the FF and OF groups. This included greater asymmetry in PSG sclerosis and greater prevalence of lateral PSG STIR hyperintensity, although these were not statistically significant at a horse level on mixed model analysis. Of the limbs contralateral to fracture, four (36%, 4/11) had confirmed incomplete fracture in the distal third metacarpal/metatarsal bone, with a further three contralateral limbs having possible incomplete fracture line or enlarged vasculature in the lateral PSG/condyle. Prodromal changes or incomplete fracture pathology have been found in contralateral limbs to fracture in many studies [12,38,39,40]. Much of the pathology in Thoroughbred racehorse fetlocks are bilateral in nature and whilst it would be logical that fractures occur in the most loaded limb, it is possible that fractures can occur in the limb with lesser pathology secondary to compensatory gait patterns and subsequent overload [14,26]. The presence of marked PSG changes in the contralateral limbs to fracture of many horses in this study further supports the value of bilateral fetlock imaging in cases of presumed unilateral fetlock related lameness in racehorses.

Lateral PSG lysis was another possible indicator of increased fracture risk (Figure 5 and Figure 9). On CT, there was a trend towards the increased likelihood of fracture (fetlock or elsewhere) in horses with lateral PSG lysis compared to the control. This may indicate a region of microdamage accumulation in the lateral PSG. PSG lysis on CT was significantly associated with high-grade sclerosis on CT and with STIR hyperintensity on MRI. The combination of lysis with surrounding sclerosis has been previously associated with increased condylar fracture risk [18,24]. In this study, this combination may be more suggestive of overall fracture risk than be specific for condylar fracture.

STIR hyperintensity in the lateral parasagittal groove has been previously associated with an increased risk of lateral condylar fracture [19]. However, the findings in this study suggest that STIR hyperintensity in this location is relatively common in horses without condylar fracture and in a population of horses in which condylar fracture is uncommon. Additionally, horses with fetlock fracture were found to be no more likely to have STIR hyperintensity in the parasagittal grooves than those without fracture. STIR hyperintensity was present in all PSGs with grade three sclerosis. Further research is needed to determine the causes of STIR hyperintensity, such as hemorrhage, edema, neovascularization or osteonecrosis in this location, so that better recommendations can be made with respect to clinical relevance.

In clinical practice, advanced imaging changes in the parasagittal grooves need to be interpreted in the context of the horse’s signalment, exercise history and presentation. In this study, there were significant age-related differences in the imaging findings. The juvenile group (two and three years old) in this study had more evidence of PSG lysis compared to the adults (older than three years). This may have been due to less bone adaptation in this group or higher bone turnover earlier in their career as the bone adapts to the stresses of training and racing. The older horses had overall more PSG sclerosis and STIR hyperintensity, possibly fitting with a higher degree of cumulative bone fatigue or better adaptation. In contrast, PSG cartilage and subchondral bone defects were not different between the age groups, suggesting that these changes are widespread in the population and may have limited clinical significance. 

PSG cartilage and subchondral bone defects were common across all groups and lateromedial distribution of SCB defects was not significantly different for reason for death or contralateral limb. There is some research to suggest that PSG cartilage and SCB defects in Thoroughbred fetlocks are developmental rather than exercise-related [32,41]. Subchondral bone and cartilage changes have been identified in the parasagittal grooves of untrained young Thoroughbreds with the microscopic appearance and large individual variance leading the authors to propose an osteochondrosis-like lesion aetiology for parasagittal groove defects [41]. It is possible that many horses have fissures or defects in this area without clinical relevance. It may also be the case that developmental defects in this area act as a stress riser or area of weakness for later fracture propagation. In foals, prominent differences in the trabecular bone between the lateral parasagittal grooves and condyles have been found and they remain present in adult horses in racing, possibly reflecting a material propensity for weakness at this site [32]. 

A proportion of the PSG defects detected on CT were thin linear regions of hypoattenuation with or without surrounding changes (Figure 8 and Figure 10). Differentiation of some of these linear defects from vascular channels was attempted histopathologically but with no success. The presence of surrounding STIR hyperintensity may indicate these cases have active remodeling or microdamage accumulation in these regions associated with early or incomplete fractures; however, care should be taken not to overinterpret STIR hyperintensity in this area, with 44% of horses in the NF group having STIR hyperintensity at this location. Conversely, lack of surrounding osseous changes and a focal tubular appearance on CT may indicate they are more likely to be blood vessels than bone fissures. These linear defects were sometimes difficult to interpret on helical CT and were usually not visualized on MRI.

We also had difficulty in interpreting the presence of sclerosis in the parasagittal grooves in some cases, as true PSG sclerosis was often difficult to differentiate from POD-related condylar sclerosis on CT and MRI (Figure 10). In these cases, it was not possible to separate sclerosis originating in the PSGs from sclerosis that had originated in the condyles and extended into the PSGs or from blending of the two. Sclerosis was also more difficult to assess on MRI when there was marked regional STIR hyperintensity. In those cases, T2W and T2*W sequences were often more useful than T1W sequences. The grade of POD present in this study was significantly associated with the grade of PSG sclerosis detected, indicating that these difficulties are likely due to the presence of concurrent POD pathology in the palmar/plantar condyles. This presents a challenge in truly assessing the clinical relevance of STIR hyperintensity and sclerosis in the PSGs when seen in combination with large POD lesions. This has previously been reported as a complication in assessing PSG sclerosis using high-resolution CT [13]. It is possible that focal PSG sclerosis and STIR hyperintensity, when detected without concurrent condylar changes, may be more clinically relevant and suggestive of bone microdamage, indicating the need for training modifications [16].

### 4.2. Imaging Techniques

Throughout this study, we assessed the comparative ability of radiography, CT arthrography and MRI to diagnose and characterize changes in the parasagittal grooves of the distal third metacarpal/metatarsal bone. By comparing certain findings and their appearance/visibility with the different imaging modalities, we aimed to gain further knowledge about the relative use of each modality for the detection of changes in the parasagittal grooves.

As hypothesized, radiography had poor detection of subchondral bone lysis and sclerosis compared to CT and MRI and false positives were common. Many of the limitations of radiography previously described in the literature were also found in this study [2,11,21,42]. Therefore, care should be taken when solely relying on this modality for diagnosis and decision-making on the presence and clinical relevance of parasagittal groove changes in racehorse fetlocks. Although the use of flexed DP radiographic views has been shown to improve detection of bone pathology in the palmar aspect of the distal third metacarpus [24,29,43], they commonly still miss small changes in the parasagittal grooves. Multiple flexed DP angles should be acquired in order to maximize the detection of lesions in different areas of the palmar/plantar aspect of the condyles. However, this does not replace the need for advanced cross-sectional imaging in many cases of suspected fetlock pathology [37,43,44].

In agreeance with previous studies, more articular cartilage and subchondral bone morphological changes were detected on CT with contrast arthrography compared to MRI [2,3,45,46]. Despite the use of low-field standing MRI rather than a higher resolution high-field MRI [47,48], the MRI findings in this study were relatively similar to CT with respect to the detection of sclerosis and subchondral bone defects. Consistent with what has been shown in previous studies [2,3,45], contrast CT arthrography was superior to low-field MRI for the detection of articular cartilage defects and subchondral bone lysis in the PSGs. MRI was better than radiography for the detection of overall pathology in the PSGs.

In this study, cartilage abnormalities were best visualized on CT contrast arthrography. This is likely due to the improved image resolution, thinner slice thickness and use of intra-articular contrast compared to MRI. On CT, the parasagittal plane was, unexpectedly, often more useful than the frontal plane for the visualization of cartilage defects. This was likely attributable to their narrow (lateromedial) but long (dorsopalmar/plantar) morphology.

There were two distinct imaging appearances of cartilage abnormalities on CT: cartilage thickening with irregularity and hypoattenuation, and cartilage loss. The cartilage irregularity, thickening and hypoattenuation that was seen on CT contrast arthrography in many of the cases in this study has previously been described in early articular cartilage damage due to collagen breakdown and cartilage swelling [46]. Cartilage loss on CT, potentially indicating a more severe full-thickness cartilage lesion, was seen less frequently in this study. The clinical relevance of cartilage loss on CT is not known but, in this study, the presence of grade two and three cartilage lesions on gross pathology was associated with the presence of SCB defects on CT.

Articular cartilage defects were harder to see with low-field MRI compared to CT contrast arthrography and the presence of cartilage lesions likely complicated the interpretation of subchondral bone defects on MRI. There were a high number of false positives and false negatives on MRI for subchondral bone defects when compared to CT. Previous studies have also found a relatively high likelihood of false positives with high and low-field MRI for cartilage and subchondral bone lesions when compared to histology [2,47,48,49]. It is likely that subchondral bone defects and grade two and three cartilage lesions in the parasagittal groove have a similar appearance on low-field MRI. Differentiation of the two pathologies may be difficult on MRI due to volume averaging artefact at the palmar aspect of the distal metacarpus and the larger slice thickness on MRI compared to CT. 

Similar to a recent study in which frontal MRI sequences were best for the detection of condylar fracture [19], the best sequence in our study for detection of subchondral bone defects on MRI was the T1W sequence in the frontal plane. However, the frontal T1W sequence also had the highest incidence of false positives for SCB defects. Fewer defects were detected on T1W transverse and condylar plane sequences; however, the likelihood of the defect being a false positive was much lower. For the condylar plane sequences, this is likely due to the orientation of the slices being more perpendicular to the articular cartilage, creating less volume averaging artefact and so better differentiation of cartilage and SCB defects.

Low-field MRI had very poor detection of subchondral bone lysis in this study, with no true positives and two false positives. It is likely that detection of lysis was not possible on low-field MRI due to the reduced image resolution compared to CT. Lysis was also sometimes challenging to diagnose on CT as the areas of lysis were often subtle and ill-defined. This suggests that if lysis is definitively detected on CT, especially in the lateral PSG, it is potentially clinically relevant. However, detection on MRI is less likely to be true.

The presence of subchondral and adjacent medullary bone sclerosis was consistently graded between CT and low-field MRI. Asymmetrical sclerosis was an important advanced imaging finding in this study and may be significant for detecting horses with increased fracture risk. Therefore, the ability to reliably detect this change on both CT and MRI is encouraging. For both CT and MRI, sclerosis was more prevalent and of a higher grade when images were viewed in the condylar plane sequence versus the frontal plane sequence and there was better agreeance between MRI and CT when the condylar plane sequence was used. This is likely due to the slice orientation being more perpendicular to the articular cartilage and thus providing a more accurate representation of the sclerosis present. For this reason, we recommend using this slice orientation when assessing CT acquisitions and adding this T1W condylar sequence to a standard racehorse fetlock MRI protocol.

### 4.3. Limitations

There are several limitations to this study, mostly centering on the use of cadaver limbs, study size and case selection.

The imaging findings in this study are from cadaver limbs, not live animals. CT and MR image quality is therefore optimized in this study due to lack of motion artefact compared to the standing horse. Cadaver distal limbs were positioned in a weight-bearing position in the magnet; however, there was very little weight through the fetlock compared to a standing horse and so limited compression of the opposing articular surfaces. Therefore, care should be taken when extrapolating the reliability and accuracy of the MRI findings to standing horses as image quality may be compromised in vivo. Equally, this study did not investigate standing CT. Standing systems are likely to have more motion artefact and lower image quality than the helical CT system used in this study [50].

All limbs were imaged with CT contrast arthrography prior to MR imaging. This resulted in all fetlock joints containing an intra-articular iodine contrast medium at the time of MRI examination. A similar previous study using high-field MRI following CTA with iodine contrast medium found the contrast was isointense to the cartilage in all MRI sequences used and did not improve cartilage defect detection [2]. Similarly, our impression from our study is that the iodine contrast did not improve our detection of cartilage defects on MRI. Interestingly, the increased volume of fluid within the joint may have improved the signal to noise ratio in the images and hence the image resolution. The use of a saline solution may have improved the detection of articular cartilage defects on MRI due to its hypointensity compared to the cartilage [51]; however, this was not the purpose of this study and CTA with the iodine-contrast medium was deemed more important.

The angle of the flexed dorsopalmar radiographic view in this study was not standardised, and multiple angles were not taken. A complete radiographic series with multiple flexed views and a very critical appraisal of the images is essential to get the most out of radiography as a diagnostic tool [43]. The potential for false positives and negatives, however, remains high with this modality.

There is some controversy about what constitutes an appropriate control group for studying musculoskeletal injury in racehorses [52,53]. Despite not being matched for age, sex or performance level, the horses in the control group in this study do fit the definition of an appropriate control: horses with the same presentation (Thoroughbred racehorses in work with death at a racetrack in south-east Queensland) that do not have the target disease (fetlock fracture). We cannot rule out, however, that some of these horses were carrying clinically significant fetlock pathology that had or had not been diagnosed prior to their death.

Front and hindlimbs were not separated for data analysis and it is not known if this pooled sampling reduced the detection of significant differences between groups.

There is likely some sampling bias in this study due to convenience sampling. However, this study still provides important information on the low-field MRI and CT imaging appearance of parasagittal groove pathology in a population of Thoroughbred racehorses in south-east Queensland with and without fracture. Further studies should aim to increase the sample size and better match the control group.

Consideration should also be made for the multicollinearity between some of the factors assessed. For example, age and reason for death are inter-related and viewing them as separate factors could be misleading. Multivariable analysis was not performed in this study and larger sample sizes would be required for statistical analysis of risk factors in future studies.

## 5. Conclusions

Changes in the PSGs of the third metacarpal/metatarsal bones of the Thoroughbred racehorse are common on CT and MRI; however, certain findings and combinations of findings are more prevalent in horses with catastrophic fractures. Rather than indicating a specific risk of fracture at the parasagittal groove, it is possible that some of these changes indicate skeletal overload and bone fatigue, suggesting an overall increased risk of fracture.

Asymmetrical sclerosis was associated with fetlock fracture and was equally visible on CT and low-field MRI. The presence of lysis in the lateral PSG was also associated with fetlock fracture; however, lysis was only detected on CT in this study, not MRI. The fetlocks contralateral to limbs with fracture often had substantial changes, highlighting the value of bilateral fetlock imaging in these horses.

PSG STIR hyperintensity was common across all groups and its clinical relevance was not clear in this study. However, the presence of PSG STIR hyperintensity on MRI was associated with the presence of lysis on CT. It was also detected on MRI in the absence of CT changes in some cases, emphasizing the need for further elucidation of what osseous STIR hyperintensity signifies in this region and its association with fracture risk.

PSG cartilage lesions were ubiquitous in this population and best detected on CT contrast arthrography; however, they are likely not clinically significant. Equally, PSG subchondral bone defects were common and not associated with a fracture but were difficult to differentiate from cartilage defects on MRI, leading to high false positives for cartilage and SCB defects on this modality. Radiography had poor detection for all PSG pathologies, even when graded as severe on the advanced imaging modalities.

## Figures and Tables

**Table 1:** Prevalence of subchondral bone sclerosis in the lateral and medial parasagittal grooves (PSG) on CT, MRI and radiography in the frontal and condylar planes in zone 1. Results are also divided into groups by reason for death (fetlock fracture FF, other fracture OF, non-fracture NF), age (adult: >3 years old, juvenile: 2- and 3-year-olds) and contralateral limb to fracture (contralateral CL, control).

	Overall	FF	OF	NF	Adult	Juvenile	CL	Control
	n (%)	n (%)	n (%)	n (%)	n (%)	n (%)	n (%)	n (%)
CT—Lateral PSG								
Frontal	53 (70%)	10 (63%)	17 (71%)	26 (72%)	19 (70%)	34 (69%)	7 (64%)	26 (72%)
Condylar	60 (79%)	10 (63%)	21 (88%)	29 (81%)	23 (85%)	37 (76%)	8 (73%)	29 (81%)
CT—Medial PSG								
Frontal	74 (97%)	16 (100%)	23 (96%)	35 (97%)	27 (100%)	47 (96%)	10 (91%)	35 (97%)
Condylar	75 (99%)	16 (100%)	23 (96%)	36 (100%)	27 (100%)	48 (98%)	10 (91%)	36 (100%)
MRI—Lateral PSG								
Frontal	34 (45%)	7 (44%)	9 (38%)	18 (50%)	13 (48%)	21 (43%)	5 (45%)	18 (50%)
Condylar	39 (51%)	7 (44%)	11 (46%)	21 (58%)	12 (44%)	27 (55%)	5 (45%)	21 (58%)
MRI—Medial PSG								
Frontal	67 (88%)	16 (100%)	19 (79%)	32 (89%)	22 (81%)	45 (92%)	10 (91%)	32 (89%)
Condylar	67 (88%)	15 (94%)	21 (88%)	31 (86%)	22 (81%)	45 (92%)	9 (82%)	31 (86%)
Radiography—Lateral PSG								
	6 (8%)	2 (13%)	0 (0%)	4 (11%)	3 (11%)	3 (6%)	4 (11%)	0 (0%)
Radiography—Medial PSG								
	10 (13%)	2 (13%)	3 (13%)	5 (14%)	6 (22%)	4 (8%)	5 (14%)	1 (9%)

**Table 2:** Prevalence of subchondral bone defects or fissures in the lateral and medial parasagittal grooves (PSG) on CT, MRI and radiography. Results are also divided into groups by reason for death (fetlock fracture FF, other fracture OF, non-fracture NF), age (adult: >3 years old, juvenile: 2- and 3-year-olds) and contralateral limb to fracture (contralateral, control).

Overall	FF	OF	NF	Adult	Juvenile	Control	Contralateral
n (%)	n (%)	n (%)	n (%)	n (%)	n (%)	n (%)	n (%)
CT—Lateral PSG							
41 (54%)	9 (56%)	14 (58%)	18 (50%)	12 (44%)	29 (59%)	18 (50%)	8 (73%)
CT—Medial PSG							
45 (59%)	10 (62%)	15 (62%)	20 (56%)	15 (56%)	30 (61%)	20 (56%)	6 (55%)
MRI—Lateral PSG							
27 (36%)	5 (31%)	11 (46%)	11 (31%)	10 (37%)	17 (35%)	11 (31%)	3 (27%)
MRI—Medial PSG							
45 (59%)	12 (75%)	14 (58%)	19 (53%)	17 (63%)	28 (57%)	19 (53%)	5 (45%)
Radiography—Lateral PSG							
12 (16%)	1 (6%)	4 (17%)	7 (19%)	5 (19%)	7 (14%)	7 (19%)	0 (0%)
Radiography—Medial PSG							
26 (34%)	9 (56%)	8 (33%)	9 (25%)	9 (33%)	17 (35%)	9 (25%)	4 (36%)

**Table 3:** Prevalence of STIR hyperintensity in the lateral and medial parasagittal grooves (PSG) on MRI. Results are also divided into groups by reason for death (fetlock fracture FF, other fracture OF, non-fracture NF), age (adult, juvenile) and contralateral limb to fracture (contralateral, control).

Location	Overall	FF	OF	NF	Adult	Juvenile	Contralateral	Control
	n (%)	n (%)	n (%)	n (%)	n (%)	n (%)	n (%)	n (%)
Lateral PSG	30 (44%)	5 (36%)	12 (55%)	13 (41%)	13 (52%)	17 (40%)	6 (60%)	13 (41%)
Medial PSG	20 (29%)	4 (29%)	7 (32%)	9 (28%)	11 (44%)	9 (21%)	3 (30%)	9 (28%)

## Data Availability

Not applicable.

## Supplementary Materials

The following are available online at https://www.mdpi.com/article/10.3390/ani11123366/s1, Table S1: Tables showing the agreeance between CT and MRI for detection of bone defects in the lateral and medial parasagittal grooves (PSG) of the distal third metacarpal and metatarsal bones. CT is considered the gold standard. Table S2: Tables showing the agreeance between CT and radiography for detection of bone defects in the lateral and medial parasagittal grooves (PSG) of the distal third metacarpal and metatarsal bones. CT is considered the gold standard. Table S3: Prevalence of cartilage lesions in the lateral and medial parasagittal grooves on gross pathology, CT and MRI. Results are also divided into groups by reason for death (fetlock fracture FF, other fracture OF, non-fracture NF), age (adult: >3 years old, juvenile: 2- and 3-year-olds) and contralateral limb to fracture (contralateral, control).
