# Supplementary material for: Imaging and Gross Pathological Appearance of Changes in the Parasagittal Grooves of Thoroughbred Racehorses
Source: Animals (Basel). 2021 Nov 24;11(12):3366. doi: 10.3390/ani11123366 (PMC8697963; doi:10.3390/ani11123366)
Supplement: Supplementary file 1 [file animals-11-03366-s001.zip › animals-1449207-supplementary.pdf]

Table S1. Tables showing the agreeance between CT and MRI for detection of bone defects in the lateral and medial parasagittal grooves (PSG) of the distal third metacarpal and metatarsal bones. CT is considered gold standard.

	No lateral PSG defect on CT	Lateral PSG defect on CT	TOTAL
No lateral PSG defect on MRI	30 (86% true negatives)	19 (46% false negatives)	49
Lateral PSG defect on MRI	5 (14% false positives)	22 (54% true positives)	27
TOTAL	35	41	76

	No medial PSG defect on CT	Medial PSG defect on CT	TOTAL
No medial PSG defect on MRI	20 (65% true negatives)	11 (24% false negatives)	31
Medial PSG defect on MRI	11 (35% false positives)	34 (76% true positives)	45
TOTAL	31	45	76

Table S2. Tables showing the agreeance between CT and radiography for detection of bone defects in the lateral and medial parasagittal grooves (PSG) of the distal third metacarpal and metatarsal bones. CT is considered gold standard.

	No lateral PSG defect on CT	Lateral PSG defect on CT	TOTAL
No lateral PSG defect on radiography	30 (86% true negatives)	34 (83% false negatives)	64
Lateral PSG defect on radiography	5 (14% false positives)	7 (17% true positives)	12
TOTAL	35	41	76

	No medial PSG defect on CT	Medial PSG defect on CT	TOTAL
No medial PSG defect on radiography	26 (84% true negatives)	24 (53% false negatives)	50
Medial PSG defect on radiography	5 (16% false positives)	21 (47% true positives)	26
TOTAL	31	45	76

Table S3. Prevalence of cartilage lesions in the lateral and medial parasagittal grooves on gross pathology, CT and MRI. Results are also divided into groups by reason for death (fetlock fracture FF, other fracture OF, non-fracture NF), age (adult: >3 years old, juvenile: 2 and 3 year olds) and contralateral limb to fracture (contralateral, control).

	Overall n (%)	FF n (%)	OF n (%)	NF n (%)	Adult n (%)	Juvenile n (%)	Contralateral n (%)	Control n (%)
CT - Lateral PSG								
	68 (85%)	15 (100%)	24 (100%)	29 (81%)	24 (78%)	44 (92%)	11 (100%)	29 (81%)
CT - Medial PSG								
	69 (86.25%)	14 (93%)	23 (96%)	32 (89%)	25 (93%)	44 (92%)	10 (91%)	32 (89%)
MRI - Lateral PSG								
	59 (73.25%)	13 (81%)	24 (100%)	22 (61%)	20 (74%)	39 (80%)	10 (91%)	22 (61%)
MRI - Medial PSG								
	59 (73.25%)	14 (88%)	21 (88%)	24 (67%)	21 (78%)	38 (78%)	10 (91%)	24 (67%)
Gross pathology - Lateral PSG								
	72 (90%)	16 (100%)	24 (100%)	32 (89%)	25 (93%)	47 (96%)	11 (100%)	32 (89%)
Gross pathology - Medial PSG								
	71 (88.75%)	15 (94%)	24 (100%)	32 (89%)	25 (93%)	46 (94%)	10 (91%)	32 (89%)
Pathology grade - Lateral PSG								
0	4 (5%)	0 (0%)	0 (0%)	4 (11%)	2 (7%)	2 (4%)	0 (0%)	4 (11%)
1	17 (21.25%)	6 (38%)	6 (25%)	5 (14%)	9 (33%)	8 (16%)	5 (45%)	5 (14%)
2	47 (58.75%)	9 (56%)	14 (58%)	24 (67%)	13 (48%)	34 (69%)	4 (36%)	24 (67%)
3	8 (10%)	1 (6%)	4 (17%)	3 (8%)	3 (11%)	5 (10%)	2 (18%)	3 (8%)
Pathology grade - Medial PSG								
0	5 (6.25%)	1 (6%)	0 (0%)	4 (11%)	2 (7%)	3 (6%)	1 (9%)	4 (11%)
1	11 (13.75%)	2 (13%)	3 (13%)	6 (17%)	5 (19%)	6 (12%)	1 (9%)	6 (17%)
2	50 (62.5%)	9 (56%)	17 (71%)	24 (67%)	18 (67%)	32 (65%)	9 (82%)	24 (67%)
3	10 (12.5%)	4 (25%)	4 (17%)	2 (6%)	2 (7%)	8 (16%)	0 (0%)	2 (6%)
